# Etiology and surgical management of pediatric acute colon perforation beyond the neonatal stage

**DOI:** 10.1186/s12893-021-01213-3

**Published:** 2021-04-26

**Authors:** Sarah Siyin Tan, Kai Wang, Wenbo Pang, Dongyang Wu, Chunhui Peng, Zengmeng Wang, Dan Zhang, Yajun Chen

**Affiliations:** Department of General Surgery, Beijing Children’s Hospital, Capital Medical University, National Center for Children’s Health, No.56 Nanlishi St, Xicheng District, 100045 Beijing, China

**Keywords:** Colon perforation, Etiology, Primary repair, Resection and anastomosis, Diverting colostomy

## Abstract

**Purpose:**

Acute colon perforation is a pediatric surgical emergency. We aimed to analyze the different etiologies and clinical characteristics of acute non-traumatic colon perforation beyond the neonatal period and to identify surgical management and outcomes.

**Methods:**

This retrospective study included 18 patients admitted with acute colon perforation and who received surgical treatment.

**Results:**

Age of patients ranged between 1 month and 15 years. Five patients swallowed foreign objects (two swallowed magnets), two had colon perforation secondary to a malignant tumor (both colorectal adenocarcinoma) and two were iatrogenic (one prior colonoscopy, one air enema for intussusception). There was one perforation due to chemotherapy and Amyand’s hernia respectively. The remaining seven patients had unknown etiologies; five of them were diagnosed with colitis. Fifteen (83.3 %) patients underwent open laparotomy, among which four attempted laparoscopy first. Three (16.7 %) patients underwent laparoscopic surgery. Fourteen (77.8 %) patients received simple suture repairs and four (22.2 %) received colonic resections and anastomosis. Four (22.2 %) patients received a protective diverting colostomy and three (16.7 %) received an ileostomy.

**Conclusions:**

There is a wide range of etiology besides necrotizing enterocolitis and trauma, but a significant portion of children present with unknown etiology. Type of surgery elected should be dependent on the patient’s etiology, disease severity and experience of surgeons.

## Introduction

Acute colon perforation is a pediatric surgical emergency and is often discussed in the context of trauma or the neonatal period, where it usually presents itself as a complication of necrotizing enterocolitis (NEC) [1]. There are few studies on colon perforation related to other etiologies or past the neonatal period, and their effect on disease onset and prognosis are unknown. Additionally, treatment for colon perforation is also seldom discussed. Colostomy, once the main method of treatment, is now secondary to primary repair [2, 3]. Analysis comparing surgical methods have been unable to determine indications for different types of primary repair among the pediatric population. In this study, we reviewed our medical records, analyzed etiologies and clinical characteristics, and identified surgical management and outcomes of children with non-traumatic colon perforation beyond the neonatal period. We aimed to provide a heightened awareness of different etiologies and their relationship to acute colon perforation, to better equip clinicians who encounter such patients. We also aimed to review different surgical managements and their respective outcomes, with the ultimate goal of outlining indications for the different surgical treatment choices.

## Method and materials

This retrospective study included 18 patients who were admitted to the Department of General Surgery in Beijing Children’s Hospital with acute colon perforation from April 2008 to April 2020. Colon perforation associated with trauma was excluded. All patients selected were between the ages of 1 month and 18 years and underwent surgery as treatment for their colon perforation. Depending on the patient’s clinical presentation, open surgery, laparoscopic surveillance with open surgery or laparoscopic surgery was elected, and either primary colon repair or colon resection and anastomosis was performed. When necessary, a stoma was created. Medical records were screened for patient history, physical examinations, surgical notes and outcomes. Follow ups were carried out via telephone interviews.

Data analysis was carried out using SPSS for Windows version 17.0. All data is presented as the median [interquartile range (IQR) first quartile–third quartile]. Categorical variables are presented using frequencies and percentages. This study was approved by the Medical Ethics Committee of Beijing Children’s Hospital, Capital Medical University (2020-Z-107) and patient informed consent requirements were waived. All methods were carried out in accordance with relevant guidelines and regulations.

## Results

There were 18 patients included in this study, 9 males and 9 females. The median age was 4.6 years (IQR 0.7–10.2 years), with 8 (44.4 %) patients younger than 2 years old. 11 patients had etiologies which varied from foreign body ingestion, malignant tumor, iatrogenic effect, chemotherapy and strangulated inguinal hernia (type IV Amyand’s hernia) (Table 1). The remaining 7 patients had unknown etiologies; 5 were diagnosed with colitis. Among these 5, 1 had undergone two prior colon biopsies at different hospitals, and both indicated colitis. Another had been receiving regular enema at home. Immunodeficiency was suspected in the third patient, but further testing was not completed per the family’s request, and NEC was suspected in the fourth patient. Patients experienced symptoms for a median of 7 days (IQR 4–10 days) before seeking medical attention at our hospital. Presenting symptoms included abdominal pain (61.1 %), fever (50.0 %), vomit (38.9 %), bloating (27.8 %), diarrhea (22.2 %), hematochezia (11.1 %), constipation (11.1 %), abdominal varicose veins (11.1 %), paleness (11.1 %) and irritability (11.1 %). Physical examinations revealed the following signs: abdominal tenderness (55.6 %), abdominal muscle guarding (44.4 %), abdominal distension (33.3 %) and rebounding pain (11.1 %).

**Table 1:** Clinical characteristics of patients with acute colon perforation

No	Age	Gender	Etiology	No. of perforation	Site (colon)	Diameter (cm)	Histopathology	Surgical procedure	Stoma formation	Draining tube	Complications	Follow-up
1	8 months	M	Bowel necrosis (Stragulated hernia)	1	Ascending	0.5	–	Suture repair + hernia repair and appendectomy	–	No	–	Good
2	9 months	F	Chemotherapy (Hepatoblastoma)	1	Transverse (Splenic region)	1.5	–	Suture repair	–	Yes	–	Good
3	12 year 7 months	M	Colorectal adenocarinoma	1	Descending	0.5	Colorectal carcinoma (moderately differeniated)	Suture repair	Colostomy	No	Tightness in chest, shortness of breath, convulsions	Died
4	15 yearr	F	Colorectal adenocarinoma	1	Sigmoid	0.5	Colorectal carcinoma (moderately differeniated)	Suture repair	Colostomy	No	Surgical wound infection	Good
5	7 year 1 months	M	Foreign object	2	Sigmoid, small	0.5	–	Resection	–	No	–	Good
6	5 year 3 months	F	Foreign object	1	Transverse (Splenic region)	0.2	–	Suture repair with laparoscopic surveillance	–	No	–	Good
7	7 year 4 months	F	Foreign object	2	Ascending, small	0.5	–	Suture repair with laparoscopic surveillance	–	No	–	Good
8	3 year 11 months	M	Foreign object (Magnet)	2	Transverse, small	1.0	–	Laparoscopic suture repair	–	No	–	Good
9	1 year 9 months	F	Foreign object (Magnet)	5	Transverse, small	1.0	–	Laparoscopic suture repair	–	No	–	Good
10	6 year 8 months	F	Iatrogenic (Colonoscopy)	1	Sigmoid	0.8	––	Suture repair	–	Yes	–	Good
11	5 months	M	Iatrogenic (Intussusception)	1	Transverse	0.5	Inflammatory granulation tissue, colitis and pericolitis	Resection	Colostomy	Yes	Pneumonia and diarrhea	Good
12	1 month	F	Unknown	1	Sigmoid	0.3	–	Suture repair	–	Yes	Surgical wound infection	Good
13	13 year 3 months	M	Unknown	1	Transverse (Splenic region)	–	Inflammatory granulation tissue, purulent pericolitis	Resection with laparoscopic surveillance	–	No	–	Good
14	12 year 7 months	M	Unknown	N	Sigmoid	–	Bowel stenosis, irregular distribution of blood vessels	Resection with laparoscopic surveillance	–	No	Fistula from bowel to abdominal wall	Good
15	11 year 2 months	M	Unknown (Colitis)	1	Sigmoid	0.5	Proliferation of mucosal and submucosal layers and mesenteric capillaries	Suture repair	Ileostomy	No	–	Good
16	1 year 6 months	F	Unknown (Enema)	1	Descending	1.5	Inflammatory granulation tissue, colitis and pericolitis	Laparoscopic suture repair	Ileostomy	No	Intestinal obstruction	Good
17	6 months	F	Unknown (Immunodeficiency)	N	Ascending, transverse	0.1	Mucosa completely detached, tissue structure destroyed	Suture repair	Ileostomy	Yes	Electrolyte imbalance, abnormal blood coaguation function, severe infection	Died
18	6 months	M	Unknown (Suspected NEC)	1	Transverse	1.0	Necrosis of intestinal wall with peripheral inflammation	Suture repair	Colostomy	No	Sepsis, fungal infection, respiratory failure	Good

**Fig. 1:**
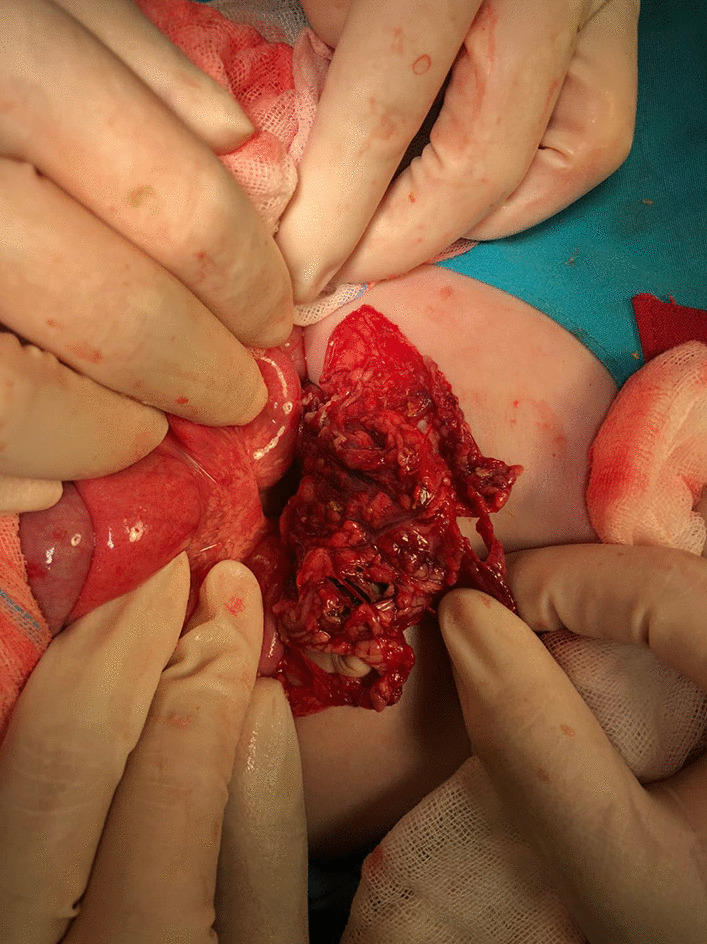
Multiple colon perforation with mesh-like appearance in a child with unknown etiology

15 (83.3 %) patients underwent open laparotomy, among which 4 attempted laparoscopy first. The remaining 3 (16.7 %) underwent laparoscopic surgery. 14 (77.8 %) patients received simple suture repairs and 4 (22.2 %) received colonic resections and anastomosis. 4 (22.2 %) patients received a protective diverting colostomy and 3 (16.7 %) received an ileostomy. Simultaneous high ligation of the hernia sac and appendectomy (due to incarcerated appendix) was performed for 1 patient and simultaneous ileus perforation repair was performed for another, according to their primary disease. The ascending, transverse, descending and sigmoid colon was affected in 3 (16.7 %), 8 (44.4 %), 2 (11.1 %) and 6 (33.3 %) patients respectively. On gross examination, it was found that 12 patients had a single perforation site, 3 patients had 2 perforation sites and 1 patient had 5 perforation sites. 2 patients had numerous perforation sites that were not identified; 1 patient’s bowel had perforations that lined the entire ascending and transverse intestine in a mesh-like appearance (Fig. 1). Biopsies were obtained for 9 patients. Histological examinations for the two patients with malignant tumors revealed moderately differentiated adenocarcinoma. One histological examination resembled NEC and the remaining 6 histological examinations all revealed nonspecific features such as inflammatory granulation tissue, colitis and pericolitis. More details can be found in Table 1.

8 (44.4 %) patients presented with post-operative complications, which included 2 surgical wound infections, 1 pneumonia and diarrhea, 1 intestinal obstruction, 1 enterocutaneous fistula, 1 sepsis and respiratory failure, 1 shortness of breath accompanied with convulsions, 1 electrolyte imbalance, abnormal blood coagulation function with severe infection. Among them, 3 (16.7 %) received re-surgeries due to surgical wound infection, gastrocutaneous fistula and intestinal ischemia of the stoma. Complications in two patients resulted in their families refusing further treatment and discharge against medical advice. Follow up revealed that they had both died. All other patients were healthy, reported normal bowel movements and did not have any other gastric perforations. The median length of follow up was 25.2 months (IQR 8.0–61.9 months).

## Discussion

Acute colon perforation is a well-recognized but rare and life-threatening problem in the pediatric population [2]. During the neonatal period, it frequently presents as a complication of necrotizing enterocolitis [4]. When injury occurs in infants and older children, it is commonly associated with blunt trauma injuries to the abdomen [2]. Among adults, inflammatory bowel disease and mechanical obstruction are the main reasons for colon perforation [5]. Spontaneous perforation of the colon is unusual, especially in children without pre-existing conditions such as Hirschsprung’s disease, inflammatory bowel disease, connective tissue disorder, lymphoma, and infective colitis [6].

Reasons for colon perforation in this study were noted as the following. First, despite literature reporting peak incidence of colon perforation due to foreign object ingestion being between 6 months to 3 years [7], of the 5 incidences, only 1 patient was younger than 2 years old. While most objects can be passed out of the alimentary tract without incident, 1–5.6 % result in colon perforation [7, 8]. We noted that all incidences of ingestion of foreign objects occurred after 2018. This is similar to a 2020 study that found recent increase in pediatric gastrointestinal tract magnets ingestion in China [8]. Second, colorectal adenocarcinoma led to 2 colon perforations in this study. The incidence of perforation among colorectal cancer is 3–10 % [9]. In the pediatric population, colorectal cancer has a significantly higher proportion of aggressive histology and is more likely to be advanced-stage at presentation [10–12]. Third, iatrogenic cases resulted in 2 colon perforations. The first was a female with Peutz–Jeghers syndrome who had received a polypectomy via colonoscopy at an external hospital. Limited pediatric studies for colonoscopies report perforation incidence at 0.01–6.7 %, whereas literature for interventional colonoscopies across all ages report a 2–3 % rate [13, 14]. A previous study on colorectal polypectomy with colonoscopy in our hospital revealed a 0.4 % perforation rate [15], falling on the lower end of other literature. Carefully performed colonoscopy can help reduce perforation incidence. The second iatrogenic patient had received a successful barium enema for intussusception 4 days earlier. Nonoperative reduction using hydrostatic or pneumatic pressure by enema is the recommended treatment, but clinicians must find a balance between aggressively attempting reductions and the risk of perforations [16, 17]. Patients whose clinical course is longer and whose bowels have housed the swollen intussusceptum for an extended period of time, thereby resulting in pressure ischemia and necrosis of bowel, would be more susceptible to perforation [18, 19]. Fourth, chemotherapy induced perforation (doxorubicin, vincristine and cyclophosphamide or cisplatin for hepatoblastoma) was another etiology. Spontaneous gastrointestinal perforation can occur in patients receiving systematic chemotherapy even without the presence of tumors [20]. A possible explanation is that chemotherapy causes weakening of tissues and rapid tumor necrosis, leading to tumor lysis and exuberant granulation which makes the bowel more susceptible to perforation [21, 22]. Fifth, one patient had a strangulated inguinal hernia with herniation of the small intestine, appendix and ascending colon (type IV Amyand hernia based on Losanoff’s classification[23]). Perforation of herniated bowel is not well documented, but Chihara et al. reported a 9.4 % rate of large bowel incarceration for inguinal hernia in adults, and a 7.5 % rate for overall bowel perforation [24].

The remaining 7 patients had unknown etiologies. Use of non-steroidal anti-inflammatory drug (NSAID), even for a short period of time, and non-typhoid Salmonella infection has been associated with spontaneous colon perforation [25, 26]. Patients with unknown etiologies in this study did not use NSAID, but their specimens were not tested for bacteria. More research is required to better understand why these children experienced spontaneous colon perforation. 5 were diagnosed with colitis prior to their colon perforation. Colon perforation is one of the most frequently encountered complications with colitis but diagnosing colitis in a child is tricky because of its many variations. Clinical presentation ranges drastically from mild symptomatic states to fulminant toxic colitis [27]. In some cases, bowel-wall thickening in imaging and histological changes of collagenous colitis are the only diagnostic clue [27, 28]. All 5 patients had pathology reports which confirmed colitis, but the specific type of colitis was not revealed [29]. Of these 5, there was one 6-month-old patient with suspected NEC. NEC diagnosis is difficult because there is no unambiguous case definition [30]. Very few published studies have been devoted to NEC in older infants. 2 case series described NEC’s prevalence in children up to 2 years old, whereas another series by Moss detailed NEC in children up to 17 years of age [31–33]. Moss maintained that NEC was the correct diagnosis despite similarities to pneumatosis intestinalis because of consistent pathological findings [33]. Definite diagnosis is based on histopathological findings of intestinal inflammation, infarction and necrosis, but clinicians primarily  use Bell’s staging criteria, which takes clinical signs, radiologic findings and laboratory data into consideration [30, 34–37]. Our patient had a history of gastroenteritis and a chief complaint of abdominal distension, vomit and hematochezia. He had shock, poor vitals, lethargy and abdominal wall erythema upon presentation, all of which support NEC [36]. His histological examination was also consistent with NEC; infarction and necrosis of intestinal wall with neutrophil infiltration and peripheral inflammation of the bowel. However, non-specificity of his presentation and lack of imaging studies prior to his surgery limit our diagnosis. Pathogenesis of late presentation NEC remains unknown, but Dagan et al. suggested that the effect of malnutrition in NEC was greater in patients beyond the neonate period [31]. Takayanagi also noted that gastroenteritis resulting in hypovolemia appeared to influence the occurrence of NEC in these patients [32].

Open laparotomy, laparoscopic surveillance with open laparotomy, and laparoscopic surgery were elected based on severity of the patient’s condition and doctors’ experience. The 4 open laparotomies with laparoscopic surveillance were performed after 2019 and the 3 laparoscopic surgeries were performed in 2020. With increased experience, we suspect that a higher percentage of future colon perforation surgeries will be laparoscopic, especially for children with single foreign body ingestion. Mattei et al. argued that laparoscopy has been routinely used in the pediatric population with excellent results, and supported its use in perforation repair [13]. Chiang and Lee similarly concluded that primary repair of perforation and peritoneal lavage using single trocar laparoscopic technique obtained good results [38]. For patients with colitis or with unknown etiology who might present with numerous perforations, laparoscopic surveillance and subsequent primary laparoscopy should first be attempted. In the more severe cases, open laparotomy with or without stoma formation is still preferred. In centers where laparoscopy experience is lacking, we recommend that surgeons practice precaution and directly perform open laparotomy. Especially when dealing with patients in critical condition, preventing further complications of the disease and deterioration of the patient is crucial. Surgeons should carefully check the bowel for other perforations and meticulously perform peritoneal lavage.

Type of surgery and primary repair was elected based on severity of the patient’s condition and doctors’ experience. Stoma formation was once the main treatment for colon perforation but with advancement of technology and surgical skills, primary repair is now more widely used [3]. Dokucu et al. suggest that primary repair could be the standard approach, particularly in children under the age of 10, as there were significantly less complications [2]. When comparing intestinal stoma versus primary repair in NEC patients, Rozeik et al. found that both options had nearly equal morbidity and mortality, but preferred primary repair because a second surgery could possibly be avoided [39]. We recommend that primary repair be the first choice of treatment and colostomies be performed only when necessary. The first and most important consideration for primary repair should be the patient’s general condition. APACHE II, SOFA, POSSUM and preoperative acute DIC scores can be used as prognostic factors for colon perforation and could aid surgeons when forming their treatment plan [40, 41]. For patients with severe conditions or who are at high risk of dying due to unstable hemodynamics and serious infection, a colostomy should be performed as a life-saving procedure. A second consideration is the condition of the anastomosis; good blood supply, tension-free bowels and meticulous technique are essential for creating a sound anastomosis [3, 42, 43]. Surgeons should ensure that the bowel surrounding the anastomosis have good blood flow, healthy tissues, clear and intact structures, and good apposition without tension [44]. Perforation size, degree of injury and devascularization of colonic wall all positively correlate with anastomotic breakdown [42]. The best candidates for primary repair are patients with minimal peritoneal contamination and good composition of bowel wall, whereas patients requiring massive resuscitation and/or with destructive colon injuries have a much higher chance of experiencing anastomotic leakage and are therefore better suited for diverting stoma formation [42]. Additionally, in patients with single foreign body ingestion, although trimming the margins of the hole before suture might be required, we suggest that simple suture repair be elected as their bowel injuries tend to be less severe [45]. For these patients, endoscopic management can be considered for defects less than 10mm and patient medical stability [46]. Insertion of draining tubes depend on severity of abdominal infection and whether peritoneal lavage can be achieved satisfactorily; they are generally encouraged in patients with multiple perforations or with leakage of bowel contents. Drains were inserted in 5 patients whose overall condition were more severe. A surgeon who is hesitant about forming a stoma can consider inserting a drainage tube [47]. The draining tube can help remove abdominal fluid collection or abscess, reduce complication rates, and act as a portal for surgeons to better understand anastomosis integrity [47, 48].

There are several strengths to this study. We have provided an in-depth retrospective analysis and discussion of the different etiologies for colon perforation in children beyond the neonatal period, an area with limited literature. Most studies available focus on gastrointestinal perforation, trauma etiologies, or the neonatal period. We have also discussed surgical management trends, the use of stoma formation and draining tubes, and provided recommendations based on different clinical presentations. Additionally, our study spans over 12 years and boasts a long follow up time. Limitations include the fact that our patients ranged from 1 month to 18 years of age. This heterogeneity combined with small patient sample did not allow us to conduct a proper subgroup analysis. Additionally, not all patients had a biopsy or have a colon specimen stored. This would have enabled us to further understand the different etiologies and how they affect colon perforation. Surprisingly, our study also did not include patients with typical etiologies such as Hirschsprung’s disease or inflammatory bowel disease, which might have led to slightly skewed results. A larger number of patients and perhaps even a meta-analysis of current literature is required for a more adequate understanding of colon perforation in the pediatric population.

## Conclusions

Acute colon perforation is a rare and life-threatening pediatric surgical emergency. There is a wide range of etiology besides neonatal NEC and trauma, like alimentary foreign body, colorectal cancer, iatrogenic injury, chemotherapy induced perforation and strangulated inguinal hernia, but a significant portion of children present with unknown etiologies. For surgery, should the patient present with colitis or with unknown etiologies, we recommend that laparoscopy should first be attempted, and that primary repair be the first choice of treatment. Surgeons who lack experience, whose patients present with poor prediction scores or overall condition, with extensive peritoneal contamination, and whose anastomosis might be compromised due to severity of the disease, should consider protective diverting stoma formation during repair. Patients with colon perforation secondary to single foreign body ingestion can receive simple suture repair endoscopically. Diverting stoma formation and insertion of draining tube have a supporting role in treatment and should be given when the patient’s abdominal infection or colonic wall injury is more severe.

## Data Availability

The data that support the findings of this study are available from the corresponding author, Yajun Chen, upon reasonable request.
